# Polarization induced control of optical trap potentials in binary liquids

**DOI:** 10.1038/s41598-018-36856-5

**Published:** 2019-01-24

**Authors:** Dipankar Mondal, Sirshendu Dinda, Soumendra Nath Bandyopadhyay, Debabrata Goswami

**Affiliations:** Indian Institute of Technology Kanpur, Kanpur, Uttar Pradesh 208016 India

## Abstract

We illustrate control of a polarized laser optical trapping potential landscape through the nonideal mixing of binary liquids. The *inherent trapping potential asymmetry* (ITPA) present in the trapping region results from the asymmetric intensity distribution in focal volume due to the high numerical aperture objective lens. Experimentally, we show that this ITPA effect can be modified and/or removed by the use of binary liquid mixtures. From our femtosecond optical tweezers experiments, we determine the topograph of the trapping potential base on the fluctuation-dissipation theorem. Additionally, the Brownian motion of the trapped bead is sensitive to the *frictional force* (FF) of the surroundings that is exerted by clusters of water and alcohol binary mixture through extended hydrogen bonding. Thus, using these two effects, ITPA and FF of the medium, we have shown that one can indeed modify the effective trapping potential landscape. Water-alcohol binary mixtures display a nonlinear dependence on the microrheological properties of the solvent composition as a result of rigid cluster formation. Volumetrically, at about 30% methanol in water binary mixture, the trapping asymmetry is minimal. In this particular binary mixture composition, the hydrophobic part of the methanol molecule is surrounded by ‘cages’ of water molecules. Enhanced H-bonding network of water molecules results in higher viscosity, which contributes to the higher frictional force. Increased viscosity decreases the degree of anisotropy due to hindered dipolar rotation. However, at higher methanol concentrations, the methanol molecules are no longer contained within the water cages and are free to move, which decrease their overall bulk viscosity. Thus, for pure solvents, experimentally measured anisotropy matches quite well with the theoretical prediction, but this fails in case of the binary mixtures due to the increased frictional force exerted by binary mixtures that result from the formation of cage-like structures.

## Introduction

Exploring intermolecular interactions is a major field of research due to its omnipresence in every aspect of natural phenomena. Parameters for quantifying and controlling such interactions are an expanding frontier, as newer experimental methodologies are developed to probe these interactions at various time and length scales^1^. The most demanding aspect of such parameterization is derived from the nonlinear interaction effects of different molecules. Such effects presently obscure molecular responses and dynamical behavior in the presence of other molecular environments, and is particularly pertinent to mixtures. At the molecular level, few mixtures are naturally isotropic, and the estimating properties of multicomponent mixtures from pure molecules is generally intractable without simplifications if they form microscopically heterogeneous regions in the matrix. Thermodynamic properties including the entropy due to mixing have an enormous impact on such interactions^2,3^ as well. Lasers have been shown to be the probe of choice for capturing nonlinearities in many systems, so, in this paper, we present the monitoring of the nonlinear behavior of binary mixtures by using optical trapping with a high repetition rate femtosecond pulsed laser.

Since their inception^4^, optical tweezers (OTs) have been advanced to measure nanometer displacements and piconewton forces on millisecond time-scales. In general, an OT uses a Gaussian beam to create a tight focus where the electric field intensity is at its maximum. Over the past two decades, this force-based technique has been widely applied to probe mechanical properties^5–8^ of single DNA, RNA, protein molecules, etc.^9–11^. The mechanical properties of these biomolecules are usually elucidated using dielectric microspheres as anchor points, and forces in the piconewton range are measured against a calibrated optical force. Furthermore, the calibration of transparent bead has been used for microrheological measurement^12–14^. We have observed that the polarization of light can change the stiffness of the calibrated bead along x and y lateral directions while the deviations in the degree of stiffness depend on the molecular properties of the trapping media. Laser polarization is thus an important parameter to control the trap stiffness due to inherent asymmetric field distribution^15–17^ around the focal plane. This phenomenon occurs when a trapping beam is focused through a high numerical aperture (NA) objective lens. In the Rayleigh regime, the induced polarization of the particle can be approximated as a point dipole, which interacts with the electric field of the trapping beam as well as the frictional forces exerted by the surroundings of the point dipole. Thus, the beam can carry either linear or angular momentum depending on the polarization state of the trapping beam. In optical tweezers, trapped particles are made to spin by transference of this angular momentum^18–21^. In our experiments, we have studied linear polarization effects on the lateral trap stiffness of a trapped polystyrene bead under the influence of ultrafast pulse train. Specifically, we have monitored a trapped particle that is suspended in binary liquid mixtures whose compositions are made to vary wherein the major contributions to the Brownian motion of the trapped particle are reduced to two: one arising from the polarizing effect of light and the other from the frictional force. The frictional force is a system dependent property that arises from the interaction between the suspended particle and the medium. Here we have shown that the properties of the light used acts as a tunable control knob for the molecular mechanical behavior of trapped particles. The landscape of the trapping potential is derived from the intensity of the incident Gaussian TEM_00_ coherent linear polarized beam, which is taken as the symmetric potential well, though in reality, the trapped potential well is intrinsically asymmetric due to diffraction through a high-NA OL. Thus, the potential landscape will have different anisotropic factors in different dipolar molecular environments, due to frictional effects. At maximum viscosity, the dipolar orientation (i.e., the movement of the trapped particle) can be restricted by the frictional force, which almost diminishes the trap stiffness asymmetry. This observation translates to being able to observe the degree of orientation control of the particle in solvents having extended hydrogen-bonding network^22^. The anisotropic field distribution due to laser polarization strongly influences the lateral stiffness asymmetric factor which may even become isotropic with the use of a high viscous polar liquid mixture as emersion solvent. Thus, the control parameters are expanded as the optical property effects like the ITPA can be modulated by judicious use of molecular interactions.

In our experiments, we have observed a significant change in the trap asymmetry due to the non-ideal behavior of the binary mixture of water and methanol^23–26^, which is reflected in micro-rheological properties, measured using absolute calibration of our femtosecond optical tweezer in the frequency domain. The femtosecond optical tweezers can trap and manipulate sub-nano to micron size particles with a steep potential well formation^27,28^ when the particles are suspended under a biologically benign condition. We have also taken advantage of forces exerted through intermolecular friction, to control the optical trapping potential. At constant temperature and laser power, for a particular trapped bead, the trap stiffness can only be changed by changing the viscosity of the surroundings. In order to delve into the viscosity effects, we made mixtures of completely miscible components (water and alcohols) and observed the effect on trapping potential landscape. We found that, altering trapping environments by changing the suspended medium changes the trapping potential in a non-linear fashion. The increased thermodynamic stability due to mixing has contributed to a reduction in the trap stiffness (κ) asymmetry^29^. The ideal behavior of the mixture property tends to fall gradually towards the higher excess of the isobaric heat capacity of mixing^30,31^. The deviations from ideal mixing behavior can be used to quantify the collective and sliding molecular motions of the mixture components in terms of excess viscosity. This information helps us to understand the control parameters of the polarization effect and ultimately the potential landscape.

Our investigation is based on observing the Brownian motion of a polystyrene bead, which is isotropic due to its spherical nature. The diffusion rate of the trapped particle, as well as the solvent molecules, is vastly affected by the viscosity^32^. The permanent dipoles of water and alcohols are considered to be linear dipoles. These dipoles can rotate at a rate that depends on their bulk viscosity and the applied external field strength. This, in turn, dictates the degree of asymmetric field distribution arising from the polarization state of light. This contactless approach of simultaneous estimation of viscosity and corner frequency (f_c_), deduced from power spectrum density (PSD)^29,33,34^ in polar solvent mixtures, enables us to calculate the lateral trap stiffness, which has information on the dipole orientation. Extended H-bonding network slows down the orientation of polar solvent molecule in the direction of laser polarization, resulting in an anisotropy minimum, which leads to the minimization of the difference of corner frequency along the x and y-axis. We have also found that at higher input trapping powers, the induced polarization exerted by the trapping field overcomes the frictional barrier imposed on the polystyrene beads by the binary mixture. So, by careful manipulation of mixture composition and input trapping power one can not only manipulate but also control the trapping potential landscape with a high degree of confidence.

## Results and Discussion

### Power spectrum method for micro-rheological measurement in binary mixtures

We have trapped 550-nm mean radius polystyrene beads coated with fluorophores using a 25-mW average power laser (Supporting Information 1). Laser power is measured before the back aperture of the trapping objective. The back focal plane position calibration is performed using a quadrant photodiode (QPD) through voltage to position conversion. A ‘sinusoidal’ response function with frequency, f_piezo_ = 50 Hz and amplitude, A = 178 nm is applied to the piezoelectric stage in the X direction, which is attached to our sample stage. The Langevin equation of trapped particle undergoing Brownian motion under such conditions (damped oscillator) can be expressed as^29,34^:

1 $$m\ddot{{\rm{x}}}({\rm{t}})+{\rm{\gamma }}\{\dot{{\rm{x}}}({\rm{t}})-{\rm{Asin}}(2{{\rm{\pi }}f}_{{\rm{piezo}}}{\rm{t}})\}+{\rm{\kappa }}x({\rm{t}})={{\rm{\zeta }}}_{{\rm{therm}}}({\rm{t}})$$

where m denotes particle mass, $\ddot{{\rm{x}}}$, $\dot{{\rm{x}}}$, x signifies time-dependent acceleration, velocity, and position, γ is the viscous drag coefficient as per Stokes’ Law, κ is the trap stiffness, and ${{\rm{\zeta }}}_{{\rm{therm}}}$ is the time-dependent random thermal force. We have saved position fluctuation data from QPD with a time resolution of 100 kHz sampling rate. Hence we can ignore the inertial effects as the characteristic inertial time scale (m/γ ≈ ns) is below our detection limit. The contribution from $\dot{{\rm{x}}}$(t) is negligible as the expectation value is a smoothly varying function. The aforementioned approximations simplified the linear equation to a form which has a general solution, when separated and solved independently. Finite time measurement with discrete Fourier transform functions has the expectation value for the one-sided PSD in the following form^34–36^.

2 $${{\rm{P}}}_{{\rm{x}}}({\rm{f}})=\langle {{\rm{P}}}_{{\rm{x}}}{({\rm{f}})}^{(\exp )}\rangle =\frac{{\rm{D}}}{{{\rm{\pi }}}^{2}({{\rm{f}}}_{{\rm{c}}}^{2}+{{\rm{f}}}^{2})}+\frac{{{\rm{A}}}^{2}}{2({({{\rm{f}}}_{{\rm{c}}}+{{\rm{f}}}_{{\rm{piezo}}})}^{2}+1)}\,{\rm{\delta }}({\rm{f}}-{{\rm{f}}}_{{\rm{piezo}}}).$$

Here D is the diffusion coefficient. The measured power spectrum in volts can be converted to a position in the X direction from the following equation^34,37,38^

3 $${\rm{\beta }}=\frac{{\rm{A}}}{\sqrt{({({{\rm{f}}}_{{\rm{c}}}/{{\rm{f}}}_{{\rm{piezo}}})}^{2}+1)}}(\frac{2{\rm{P}}}{{{\rm{t}}}_{{\rm{msr}}}}){{\rm{\zeta }}}_{{\rm{therm}}}({\rm{t}})$$

**Figure 1:**
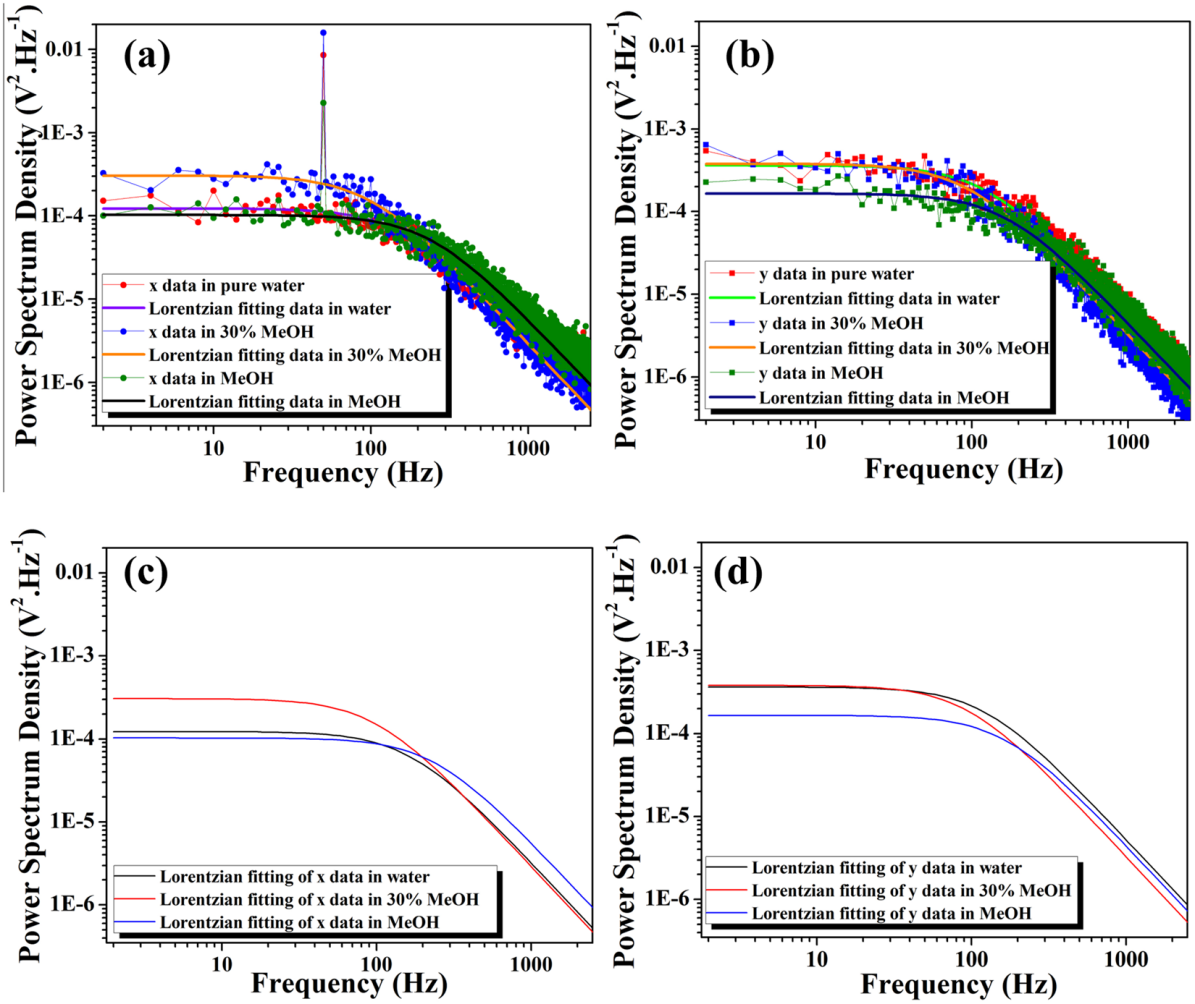
Power spectrum density for 550 nm radius fluorophores coated polystyrene bead in Water, 70:30 volume proportions of water-methanol mixture and in pure methanol of (**a**) x-axis data (Scatter points) with its respective Lorentzian fitted data (Solid line); (**b**) y-axis data (Scatter points) with the respective Lorentzian fitted data (Solid line); (**c**) Lorentzian fitting of the experimental x-axis data; and (**d**) Lorentzian fitting of the experimental y-axis data.

Here β is estimated in nm/mV, P is actual height of spike after eliminating thermal background due to Dirac delta function: δ(f-f_piezo_) experimentally created and t_msr_ is 0.5 second in our experiment. Our experimental data is fitted to the first term of the right-hand side of the Eq. 2, which is a Lorentzian. Before analysis, x and y-axis data points are de-correlated using MATLAB® programming. The fitting parameter for individual measurements gives the corresponding calibration factor β. This calibration factor can be used to evaluate the absolute viscosity around the trapping bead. We have used a thick cover glass sample chamber with thickness above 100 µm to rule out surface force-based viscous effects. Water-methanol mixtures used for our experimental investigation were prepared by the addition of alcohol by volume content of 0-100% in steps of 10% with additional data at 5% volume mixtures. Due to the very low absorption^39,40^ at 780 nm of the solvent media used, the temperature rise is considered to be negligible. Viscosity is measured with absolute calibration by the PSD method. The complete miscibility of two solvents has a marked effect on the two-photon fluorescence (2PF) (Supporting Information 2). An increase in methanol volume leads to an increase in 2PF, as the quantum yield (Φ) of Rhodamine 6 G in Methanol (0.92) is higher than that of water (0.79). Our first observed result is that, at viscous maxima (30% methanol by volume in H_2_O-MeOH mixture), the difference between the corner frequency in x and y-axis is minimized. This was measured using the power spectrum method (Fig. 1 and Supporting Information 3) (Table 1). As a result, the calculated asymmetry of trap stiffness is minimized in this case.

**Table 1:** Trapped bead parameter and relative trap stiffness.

Solvent used	f_x_ (Hz)	f_y_ (Hz)	κ_asym_ = (1 − κ_x_/κ_y_)	D (nm^2^/μs)	η (mPa.s)
Water	163 ± 10	123 ± 3	−0.33	0.488	0.894
5%	145 ± 1	115 ± 1	−0.26	0.384	1.135
10%	132 ± 2	117 ± 2	−0.13	0.331	1.314
20%	101 ± 1	90 ± 1	−0.10	0.291	1.497
30%	98 ± 2	93 ± 2	−0.06	0.270	1.615
40%	111 ± 1	96 ± 1	−0.15	0.294	1.482
50%	94 ± 2	79 ± 1	−0.20	0.337	1.293
60%	113 ± 1	93 ± 1	−0.21	0.368	1.184
70%	121 ± 1	99 ± 1	−0.22	0.425	1.028
80%	159 λ± 3	129 ± 2	−0.23	0.500	0.872
90%	197 ± 2	152 ± 1	−0.30	0.557	0.783
MeOH	239 ± 14	166 ± 2	−0.44	0.726	0.600

**Figure 2:**
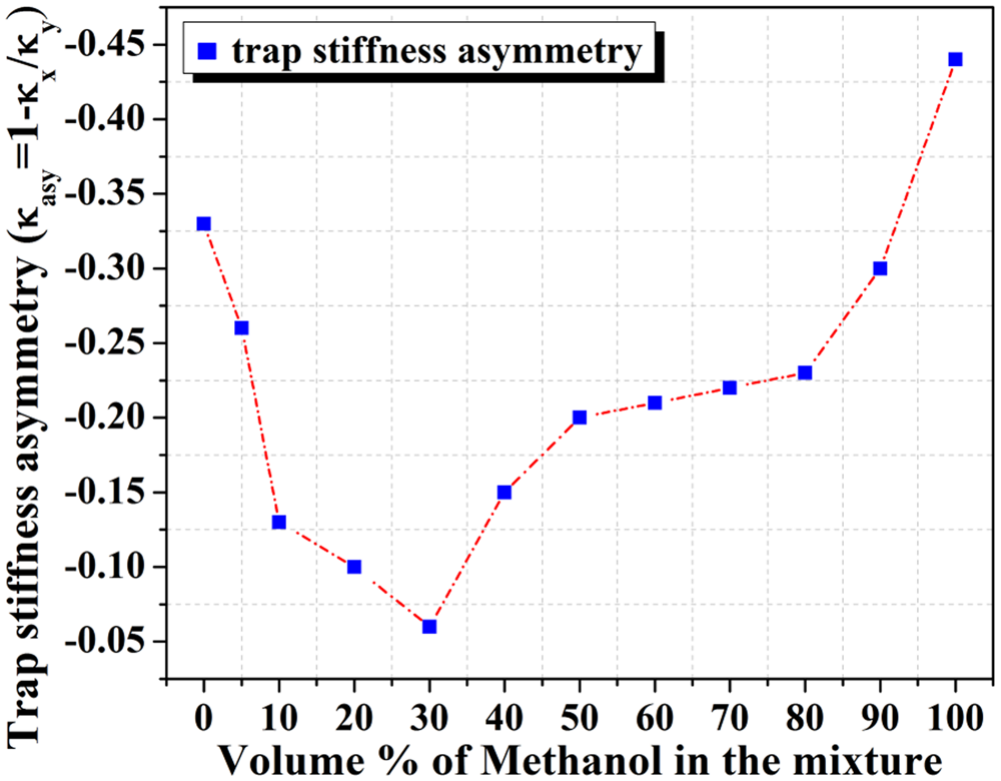
Experimentally measured trap stiffness asymmetry versus different volume proportion of water-methanol binary mixtures.

**Figure 3:**
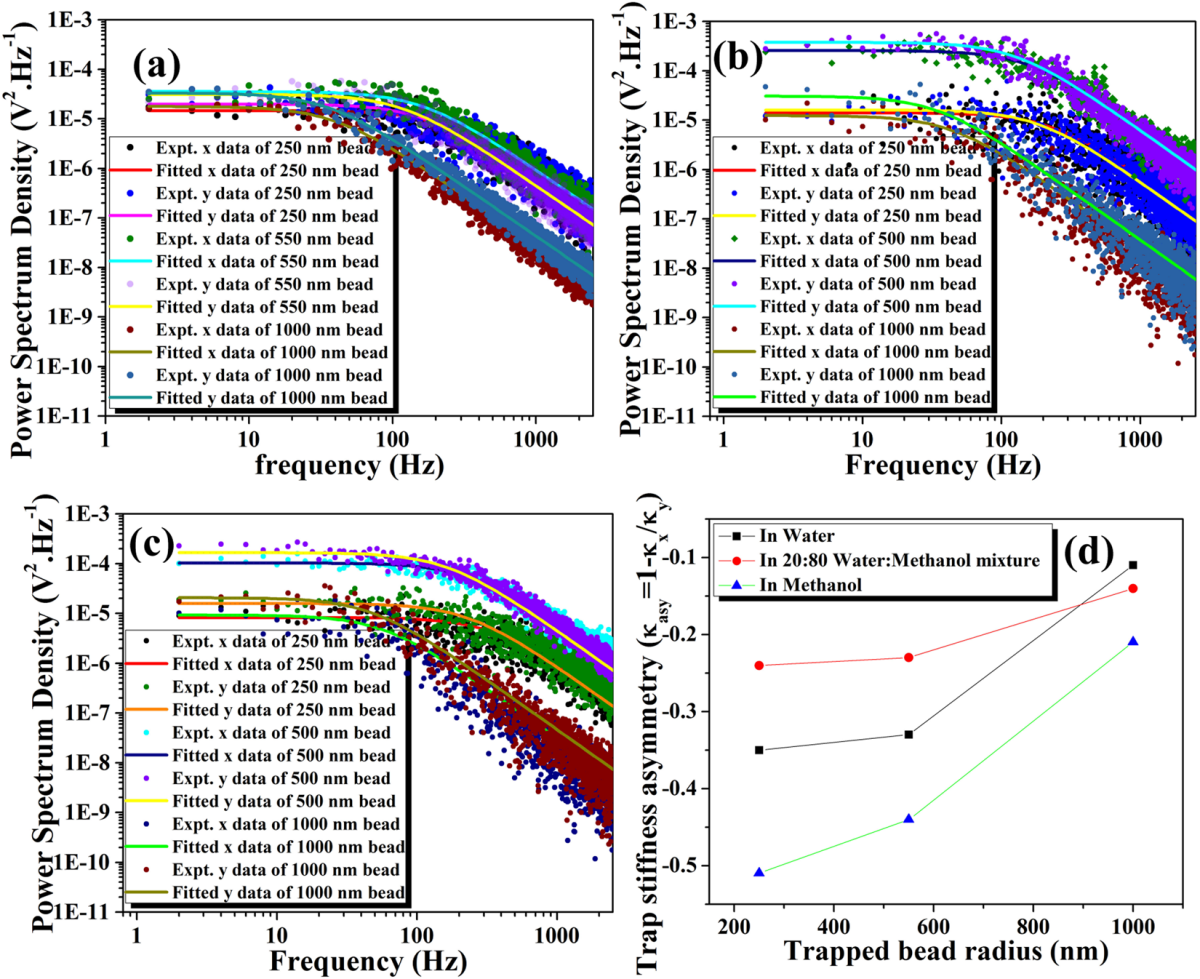
Power spectrum density (Scatter points representing experimental data) and the respective Lorentzian fitted data (Solid line) for 250 nm, 550 nm and 1000 nm radius polystyrene bead in (**a**) Water (**b**) Methanol (**c**) 80% MeOH and (**d**). The size-dependent trap stiffness asymmetry in three different solvents.

At the same time, the maximum asymmetry is observed in pure methanol (Fig. 2). The input power dependent study in the maximum viscosity regime (a situation corresponding to minimum anisotropy) shows that the trapping potential asymmetry is almost negligible in the lateral direction when input power is reduced. However, at higher laser powers, corresponding to higher field strengths, the introduced polarization asymmetry overcomes the frictional forces imposed by the network structure of the binary mixture. This counteracts the intensity distribution dependent asymmetry at the low input field strength (Supporting Information 4). We have also performed a size-dependent study to indicate changes in the viscous effects at different length scales. It is expected that as the surface area of the particles increase, they experience more frictional force^41,42^, resulting in a decrease in the trap stiffness asymmetry. We have measured the corner frequency of the trapped particle for 250 nm, 550 nm and 1000 nm radius beads in the lateral direction. The size-dependent measurements have been performed in three different solvents: water (Fig. 3a), methanol (Fig. 3b) and 80% methanol in the binary mixture (Fig. 3c). The 80% binary mixture has been chosen due to the fact that, in-spite of viscosity values which are similar to water (80% mixture has η = 0.872, water has η = 0.894), it exhibits very distinctive solvent behavior which cannot be simply ascribed to it’s pure component properties. From this study, we have determined that, between the 250 nm and the 550 nm bead, the trapping stiffness asymmetry is similar, with the 250 nm having a slightly higher asymmetry due to a decrease in surface area. The effect of viscosity is dominates observations for the 550 nm and 1000 nm radius trapped beads for all three solvents (Supporting Information 5). For 1000 nm trapped beads the trapping stiffness asymmetry decreases drastically, as observed in Fig. 3d. The asymmetry factors are inversely proportional to the size of the beads because higher radius particles have higher surface area and thus experience higher frictional forces.

### Electromagnetic propagation through a high numerical aperture

We demonstrate the appearance of trap asymmetry at the focal region, which we assign to the diffraction due to the high NA objective lens. From this, we expand and analyze our first experimental finding, which is the removal of trap asymmetry at the certain compositions of the binary mixtures (at low input laser power). Whenever a high NA OL (NA > 0.7) is used to focus a beam of light, the intensity distribution at the focal volume depends on the polarization state, spatial shape of the input beam and refractive index of the mediums involved. This method is known as Vectoral Imaging^43,44^. The intensity distribution of the beam at the focal region is represented by Debye-Wolf Integral which may be written as follows (in a homogeneous refractive index medium)

4 $$\begin{array}{lll}\overrightarrow{E}(x,y,z) & \propto  & {\int }_{0}^{\alpha }{\int }_{0}^{2\pi }\overrightarrow{E}(\theta ,\varphi )\,\exp [ik(x\,\sin \,\theta \,\cos \,\varphi +y\,\sin \,\theta \,\sin \,\varphi \\  &  & +\,z\,\cos \,\theta )\sin \,\theta d\theta d\varphi \\ \overrightarrow{E}(\theta ,\varphi ) & = & A(\theta ,\varphi )B(\theta ,\varphi )\overrightarrow{P}(\theta ,\varphi )\end{array}\}.$$

The electric field distribution after the exit pupil of the objective is due to three separate terms, amplitude A(*θ,ϕ*), apodization factor B(*θ,ϕ*) and polarization state $\vec{P}$(*θ,ϕ*) of the incident beam (the objective is symmetric around *θ* and *ϕ*, see Fig. 5A of Supporting Information 6. A Gaussian beam with x-polarization as an input creates an asymmetry in the intensity distribution at the focal plane. As the scattering gradient forces depend on the field intensity at the focus, asymmetric intensity distribution will effectively change the force constant along the relative axis. For a dielectric particle at the Rayleigh regime, the total force^15,45–47^ depends on both the scattering and gradient forces. Finally, the force constant κ_i_ at the trapping position is obtained according to the following equation

5 $${{\rm{\kappa }}}_{{\rm{i}}}={\partial }_{{\rm{i}}}{{\rm{F}}}_{{\rm{i}}}{({{\rm{x}}}_{{\rm{i}}})|}_{{{\rm{x}}}_{{\rm{i}}0}}={\partial }_{{\rm{i}}}\,[{{\rm{F}}}_{{\rm{i}},{\rm{grad}}}({{\rm{x}}}_{{\rm{i}}})+{{\rm{F}}}_{{\rm{i}},{\rm{sca}}}({{\rm{x}}}_{{\rm{i}}})]{|}_{{{\rm{x}}}_{{\rm{i}}0}}.$$

Here, F_i,grad_(x_i_) is the gradient force and F_i,sca_(x_i_) is the scattering force at the (0, 0, z_0_) trapping position. The scattering force decreases the trap stiffness in the lateral direction but increases the stiffness in axial direction^15^. So, the trapped bead will feel the asymmetric potential due to this asymmetric intensity distribution. As the intensity is higher along the x-axis due to polarized induced asymmetry, the trap stiffness will be higher along x-direction compared to that of Y-direction.

In our experimental setup, there are three layers in the sample chamber, as depicted in Fig. 5B of Supporting Information 6. The first layer is the immersion medium layer (in our case, oil), the second layer is the coverslip, and the third layer is the medium layer within which the trapped object is suspended (water, methanol or the water-methanol binary mixture). For practical purposes, the oil immersion medium and coverslip layers are usually indistinguishable. Depending on the level of refractive index mismatch, there is a significant shift in the geometrical focusing point. The introduction of this refractive index mismatch in the system is quantified by factoring in spatial aberrations and fractional transmittances in the Debye-Wolf integral and maybe now represented as^48,49^

6 $$\overrightarrow{E}(x,y,z)\propto {\int }_{0}^{\alpha }{\int }_{0}^{2\pi }(\begin{array}{c}A({\theta }_{1},\varphi )B({\theta }_{1},\varphi ){\overrightarrow{P}}_{2}({\theta }_{1},{\theta }_{2},\varphi )\\ \exp [ik({n}_{1}x\,\sin \,{\theta }_{1}\,\cos \,\varphi +{n}_{1}y\,\sin \,{\theta }_{1}\,\sin \,\varphi +{n}_{2}z\,\cos \,{\theta }_{2})]\\ \exp [ik({\rm{\Phi }}({\theta }_{1},{\theta }_{2},\varphi )]\end{array})\,\sin \,{\theta }_{1}d{\theta }_{1}d\varphi $$

**Figure 4:**
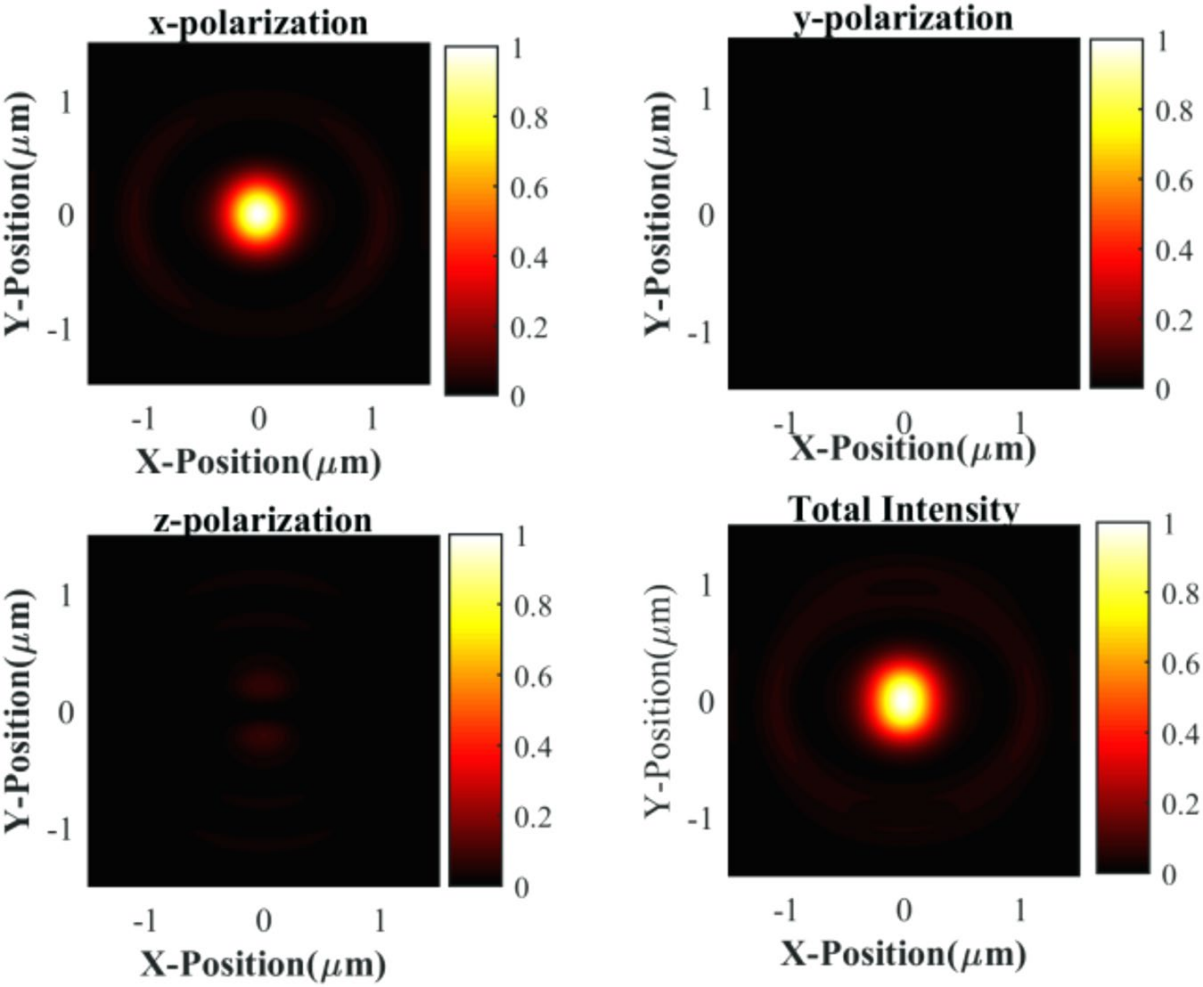
Relative intensity distribution in the focal plane (Transverse) for a Gaussian-shaped beam is represented. Other parameters are NA = 1.4 oil immersion, 780 nm wavelength, and x-linear polarization. Intensity is asymmetrically distributed in the X-Y plane.

**Figure 5:**
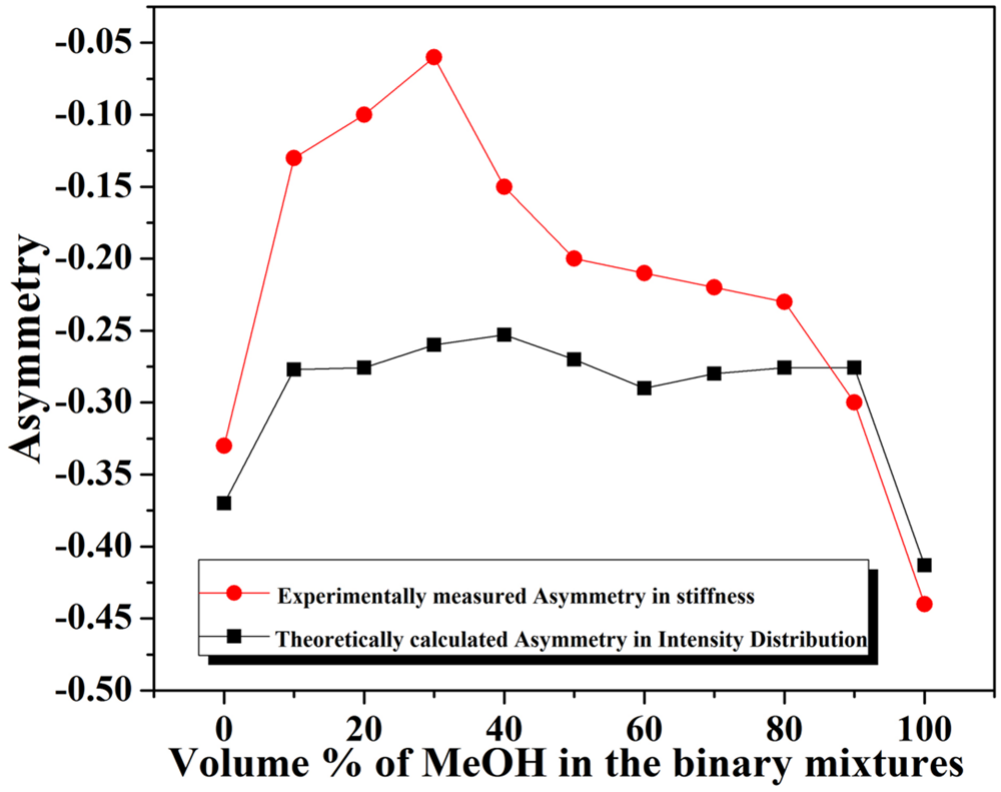
Comparative measurement of asymmetry of stiffness (Red) and intensity distribution (Black) in the focal plane (Transverse) for a Gaussian-shaped beam is represented. Other parameters are NA = 1.4 oil immersion, 780 nm wavelength, and x-linear polarization. Intensity is asymmetrically distributed in the X-Y plane.

Here n_1_ is refractive index medium, and water medium is considered as second refractive index medium, n_2_. (The n_1_ = n_2_ case arises when the medium is homogeneous). The intensity distribution for water, as the second medium, at the focal region is given by Fig. 4. We also calculated the intensity asymmetry for a different binary mixture using this method. We found that the intensity distribution asymmetry remains almost nearly constant for all the binary mixture given in Table 2. These findings contradict our experimental results, where we found that at sufficiently low input laser power the trapping asymmetry almost vanishes at 30% methanol in water mixture. As measured asymmetry changes arise entirely from solvent mixing properties and not from intensity distributions, it is solely dependent on molecular properties at the lower input power regimes. Our theoretically calculated asymmetries in the X-Y plane (trapping region) for pure water and pure methanol are −0.41 and −0.37 whereas, experimentally measured trapping potential asymmetries in the X-Y plane are −0.44 and −0.33 respectively (Fig. 5). These values match well, but for the binary mixtures, the asymmetry factor differs considerably between experimental measurements and theoretical predictions given in Table 2 due to the formation of complex structural networks by the components of the mixture at molecular levels as explained above.

**Table 2:** Comparison between our experimental and theoretical data.

Solvent used	Refractive Index^58^	Experimental κ_asym_ = (1 − κ_x_/κ_y_)	Asymmetry Intensity distribution in the X-Y plane
Water	1.3326	−0.33	−0.370
5%	1.3364	−0.26	−0.277
10%	1.3387	−0.13	−0.276
20%	1.3396	−0.10	−0.260
30%	1.3394	−0.06	−0.253
40%	1.3389	−0.15	−0.270
50%	1.3379	−0.20	−0.290
60%	1.3360	−0.21	−0.280
70%	1.3331	−0.22	−0.276
80%	1.3302	−0.23	−0.276
90%	1.3264	−0.30	−0.413
MeOH	1.3326	−0.44	−0.370

### The interaction between applied fields with molecular dipole induce the trapping asymmetry

In the following paragraphs, we will try to demonstrate qualitatively, how the network formation counteracts the effect of the asymmetric intensity distribution. Binary mixtures of polar molecules are randomly oriented without the influence of any external field, but when an external coherent linear polarized light field is applied, a torque is exerted on the permanent dipoles. This effect induces them to orient themselves in the direction of the applied field. The friction occurring within the liquid is due to its specific viscosity, which quantifies a direct measurement of the friction between the rotating spherical dipole and its neighboring liquid molecules. Particles suspended in more viscous liquids will tend to exhibit dipole losses at considerably lower frequencies. The intrinsic value of this relaxation time^50,51^ of a dipole, rotating in a viscous continuum, is represented by a time constant τ = ξ/(2k_B_T). Where ξ is the friction constant of rotating molecular sphere^52,53^ of radius r, which is given by ξ = 8πηr from Stokes law in a liquid media with viscosity η.

The expectation value of a dipole vector in the direction of an applied field having a permanent dipole µ, can be given in terms of the Boltzmann statistics at time t as^50,51^

7 $$\langle {{\rm{\mu }}}_{{\rm{t}}}\rangle =\frac{{\int }_{0}^{{\rm{\pi }}}{\rm{G}}({\rm{\tau }})\,{\rm{\mu }}\,\cos \,{\rm{\varphi }}\,(\sin \,{\rm{\varphi }}){\rm{d}}{\rm{\varphi }}}{{\int }_{0}^{{\rm{\pi }}}{\rm{G}}({\rm{\tau }})\,(\sin \,{\rm{\varphi }}){\rm{d}}{\rm{\varphi }}};\,{\rm{Here}}\,{\rm{G}}({\rm{\tau }})={{\rm{Ae}}}^{(\frac{{{\rm{\mu }}{\rm{E}}}_{{\rm{r}}}\cos {\rm{\varphi }}}{{{\rm{k}}}_{{\rm{B}}}{\rm{T}}})}.$$

Here ϕ is the inclination angle between dipoles; E_r_ is the directing field acting on the molecule (Supporting Information 7) and A = (n^2^ + 2)/2 with n being the refractive index. Equation 7 can be solved and expressed by the following equation

8 $$\langle {{\rm{\mu }}}_{{\rm{t}}}\rangle =(\frac{{{\rm{\mu }}}^{2}{{\rm{E}}}_{{\rm{r}}}}{3{{\rm{k}}}_{{\rm{B}}}{\rm{T}}})\exp (\frac{2{{\rm{k}}}_{{\rm{B}}}{\rm{T}}}{{\rm{\xi }}})$$

According to the theory of dielectric polarization, the polarization of a liquid can be expressed as^54,55^ [P] = (4π/3)N_A_{α + (gμ^2^/3k_B_T)}. Here α is optical polarizability, µ is molecular dipole moment, and $\bar{{\rm{\mu }}}$ is the sum of molecular dipole and the average electric moment induced by the molecule in its environment by hindering the rotation of its neighbors relative to itself. In the hydrogen bonded H_2_O-MeOH mixture, the Kirkwood correlation factor g is introduced to simplify the µ.$\bar{{\rm{\mu }}}$ term to gµ^2^. For intermolecular H-bonding between water and alcohol: g = 1 + 2fcos^2^(θ/2) with H-O-C angle as 105 °, and f can be expressed as^55,56^

9 $${\rm{f}}=\frac{({{\rm{\mu }}}_{{\rm{H}}}+{{\rm{\mu }}}_{{\rm{R}}})({{\rm{\mu }}}_{{\rm{H}}}+{{\rm{\mu }}}_{{\rm{R}}}\,\cos \,{\rm{\theta }})}{({{\rm{\mu }}}_{{\rm{H}}}^{2}+{{\rm{\mu }}}_{{\rm{R}}}^{2}+2{{\rm{\mu }}}_{{\rm{H}}}{{\rm{\mu }}}_{{\rm{R}}}\,\cos \,{\rm{\theta }})}$$

where µ_H_ (1.6 D) and µ_R_ (0.7 D) are the components of the dipole moment of the alcohol molecule ROH (R= CH_3_–) along the O-H and O-C axes. Corrections can be made to Equation 8 to support our experimental results.

Our experimental results indicate that, at maximum viscosity (30% methanol in the water-methanol mixture), the asymmetry in trap stiffness is minimized due to the effect of intermolecular frictional forces arising from the extended hydrogen bonding^24,57^ between the solvents, water and methanol. Water–alcohol binary mixtures exhibit a nonlinear dependence on the micro-rheological properties of the solvent components as a result of rigid cluster formation, which slows down the Brownian motion of the trapped particle, resulting in a decrease in diffusion coefficient (Supporting Information 8). Volumetrically, at about at thirty percent water and methanol binary mixture, the hydrophobic part of the methanol molecule remains surrounded by ‘cages’ of water molecules. Enhanced H-bond networks of water molecules translate into higher viscosities and lower diffusion coefficients. At high methanol concentrations, the methanol molecule is not bound by water cages and is free moving, which decreases the viscosity of the media. Rigid H-bonding networks hinder the orientation of polar solvent molecules in the direction of laser polarization, resulting in minimum anisotropy.

At maximum viscosity, the solvent molecules around the trapped bead have a rigid structure, so the dipole rotation along polarization direction is hindered, as is expected from Eq. 8 (〈μ_t_〉 ∞ exp ((2k_B_T)/ξ)). Here viscosity plays a major role in the orientation along the applied external field. However, when the applied field strength is increased, polarization effects dominate the molecular rotational hindrance effects again, so that they align perfectly along the direction of the applied field, which is our second observation. This is because, at constant viscosity, the external field strength dictates the induced polarization (〈μ_t_〉) ∞ E_r_. The power dependent study (Fig. 4a–g in Supporting Information 4) of trap stiffness (κ = 2πγf_C_), also have asymmetric relations along x and y-axis, which is evident from the differences in corner frequency (f_C_) data along the x and y-axis (Fig. 4h in Supporting Information 4). Trap stiffness is linearly proportional to the corner frequency at constant power, constant viscosity, and a fixed sized particle. Thus, at high power regimes, the laser polarization effects become a predominant parameter to dictate the trap stiffness asymmetry.

## Materials and Methods

**Figure 6:**
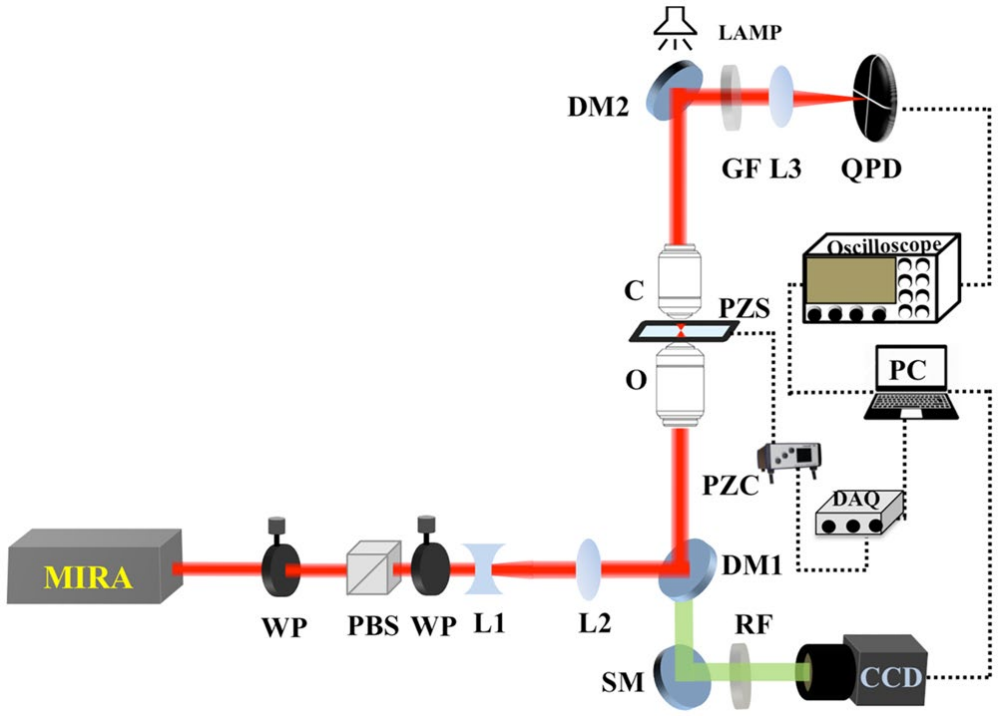
Schematic diagram of our experimental femtosecond optical tweezers set up. WP: Half-wave plate; PBS: Polarizing beam splitter; L1: Concave lens; L2: collimating convex lens; DM: Dichroic mirror; O: Objective lens; PZS: Piezoelectric Sample stage; PZC: Piezoelectric controller; DAQ: Data acquisition card; C: Condenser lens; GF: Green filter; L3: Focusing lens; QPD: Quadrant photodiode; SM: Silver mirror; RF: Red filter; CCD: Camera (Charge-coupled device) PC: Personal computer.

Our femtosecond optical tweezers set up is shown in Fig. 6 and Supporting Information 9. The laser source, used in an inverted microscope geometry, is the mode-locked Ti-Sapphire laser (MIRA-900F pumped by Verdi-V5, Coherent Inc.). The experiments presented here were performed by using femtosecond laser pulses, centered at 780 nm wavelength with a repetition rate of 76 MHz. The pulse width of 150 fs is used for optical trapping. A commercial oil immersion objective (UPlanSApo, 100X, 1.4 NA, OLYMPUS Inc. Japan) was used to achieve the diffraction-limited spot by tight focusing; simultaneously, the forward scattered light was collected at the back focal plane of another oil immersion objective (60X, PlanAapo N, 1.42 NA, OLYMPUS Inc. Japan) and focused onto a quadrant photodiode (QPD) (2901, Newport Co. USA). The QPD output was then fed to a digital oscilloscope (Waverunner 64Xi, LeCroy USA), which is interfaced with a personal computer through a GPIB card (National Instruments, USA). Data was collected for processing via LabVIEW. Commercially available fluorophores coated polystyrene beads (with concentration 2.7 × 10^10^ particles/ml), suspended in water, were purchased from Life Technology, USA (F8820, Lot number 30724 W, Currently Thermo Fisher, USA). The stock solution was diluted further to achieve a sub-nanomolar concentration for the trapping experiments and was well-sonicated for immediate use. We prepared the sample chamber by placing a coverslip 22 × 22 mm No. 1 over 24 × 50 mm No. 0 cover glass, separated by spacers of double-sided sticky tape. The sample chamber was then placed on a piezoelectric stage (NSP3, Newport Co. USA), which is operated and controlled through a Piezo controller (NSP3, Newport Co. USA) connected to a personal computer via a NI DAQ card 6212 (National Instruments, USA). The DAQ is used to provide a sinusoidal response function to the piezoelectric stage for voltage calibration. Video of the trapping event (see Supporting Media File) were captured using a CCD camera (350 K pixel, e-Marks Inc. USA). For bright field illumination, we have used a flash-light. The trapping power of the laser was measured with a power meter (FieldMate, Coherent USA), as well as a calibrated silicon amplified photodiode (PDA100A-EC, Thorlabs USA). The data analysis procedure is elaborated on in the Supporting Information 10.

## Supplementary information

Supplementary Info

Trapping in Pure Water

Trapping in Binary Mixture

Trapping in Pure Methanol
