# Supplementary material for: Polarization induced control of optical trap potentials in binary liquids
Source: Sci Rep. 2019 Jan 24;9:700. doi: 10.1038/s41598-018-36856-5 (PMC6345749; doi:10.1038/s41598-018-36856-5)
Supplement: Supplementary file 1 — Supplementary Info [file 41598_2018_36856_MOESM1_ESM.docx]

Supporting Information

Polarization induced control of optical trap potentials in binary liquids

Dipankar Mondal, Sirshendu Dinda, Soumendra Nath Bandyopadhyay, Debabrata Goswami*

Indian Institute of Technology Kanpur, Uttar Pradesh 208016, India

*Correspondence to: [dgoswami@iitk.ac.in](mailto:dgoswami@iitk.ac.in)

**Supporting Information : 1**


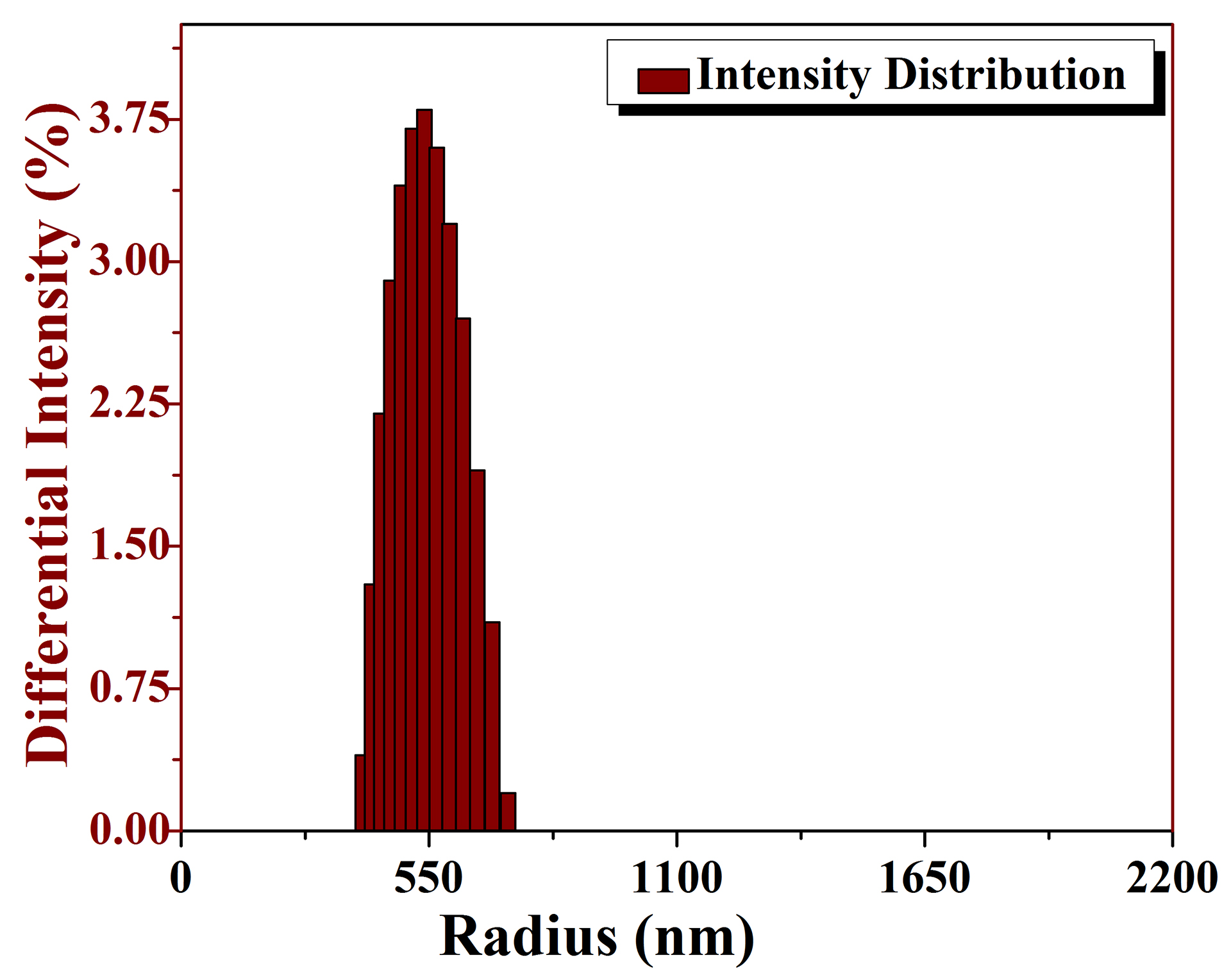


**Figure 1:** Measured differential light scattering (DLS) through our buffered polystyrene beads sample solution showing the average particle distribution size to be 550 nm.

**Supporting Information : 2**





**Figure 2:** Two photon fluorescence of Rhodamine 6g at a concentration 5×10^-5^ water-methanol binary mixture at different volume proportion.

**Supporting Information: 3**


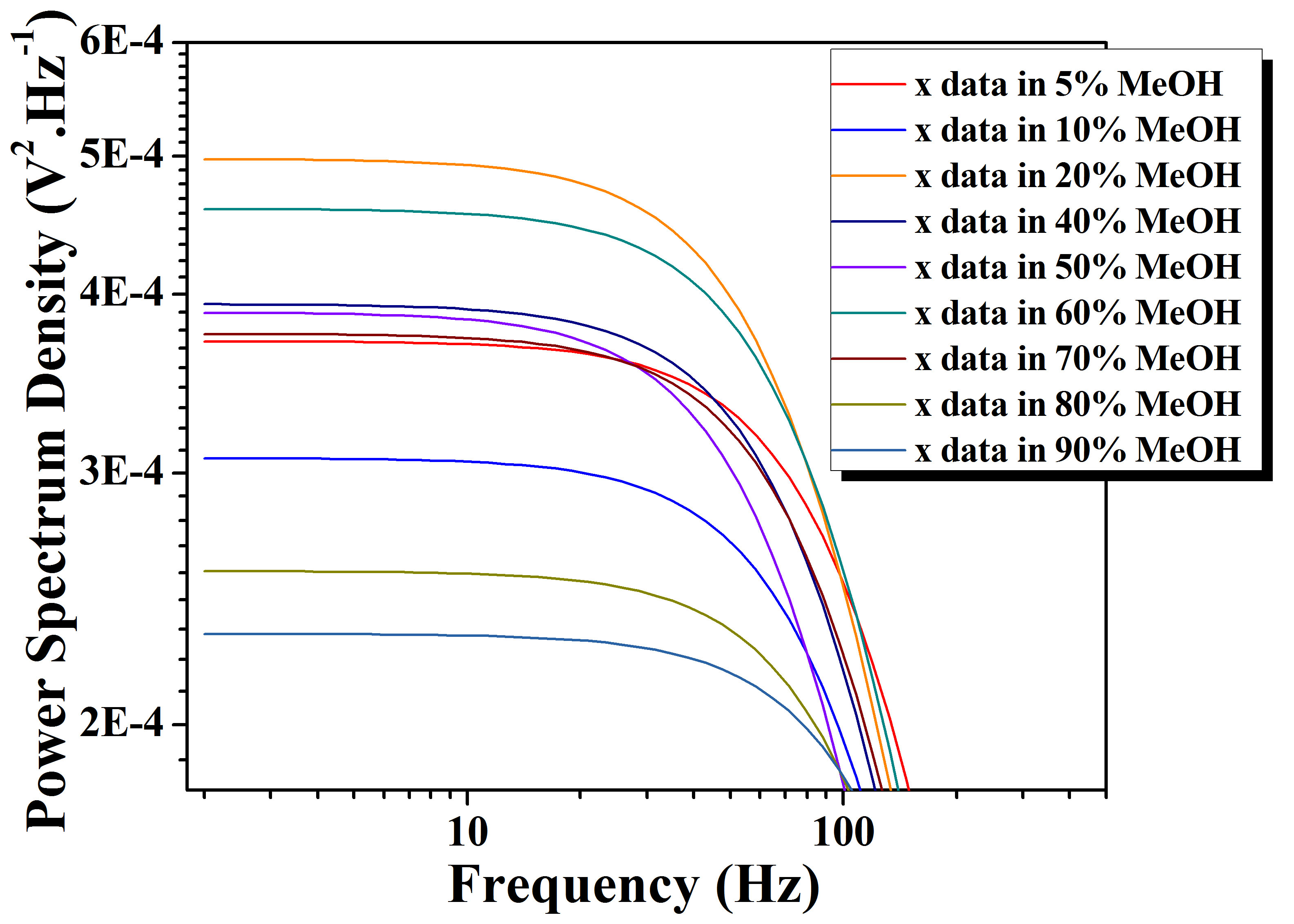

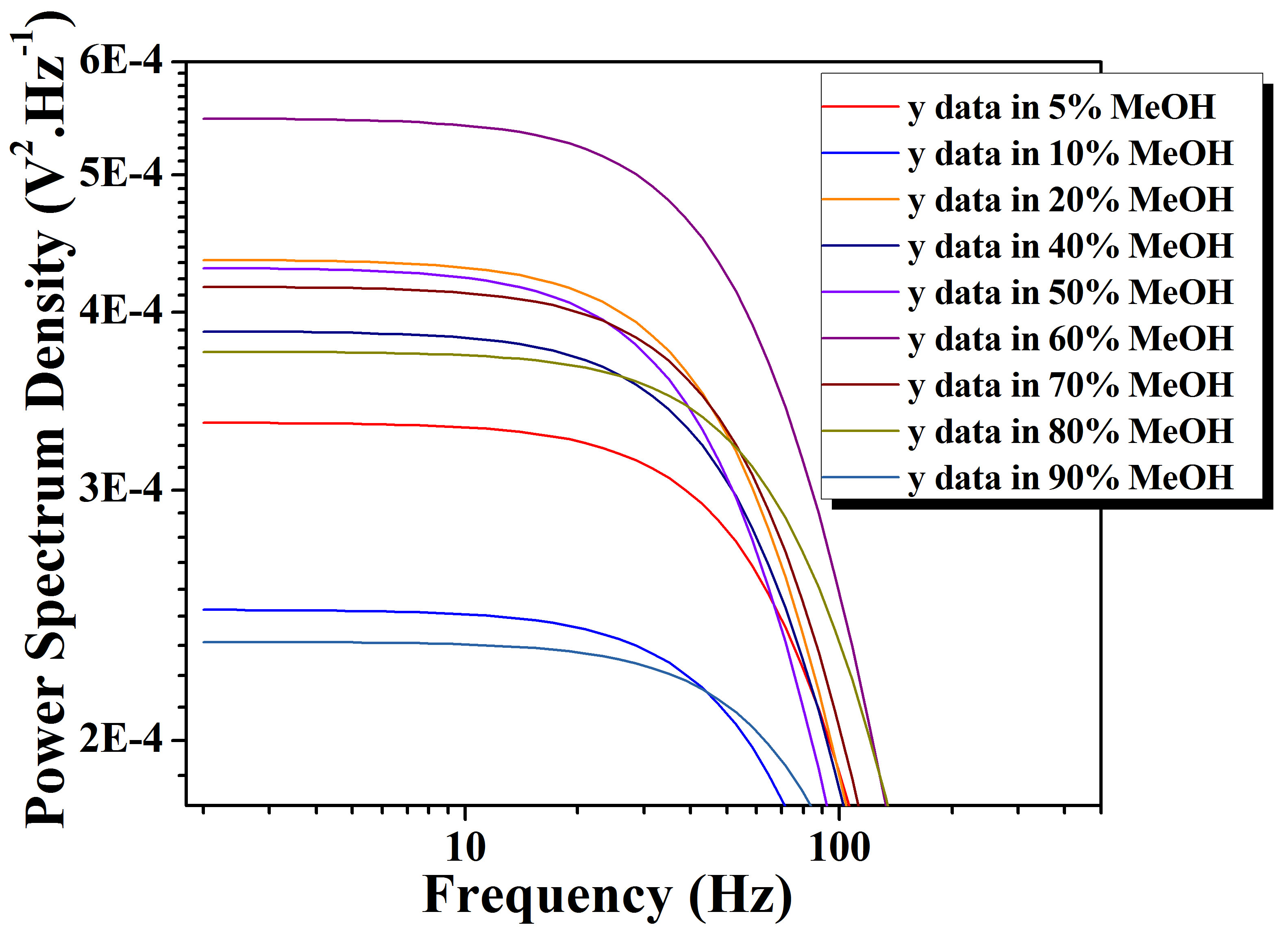


**(a)**

**(b)**

**Figure 3:** Lorentzian fitting of power spectrum density for 550 nm radius fluorophore coated polystyrene bead in water-methanol binary mixture at different volume proportion as shown in the figure (a) x axis data (b) y axis data

**Supporting Information 4**


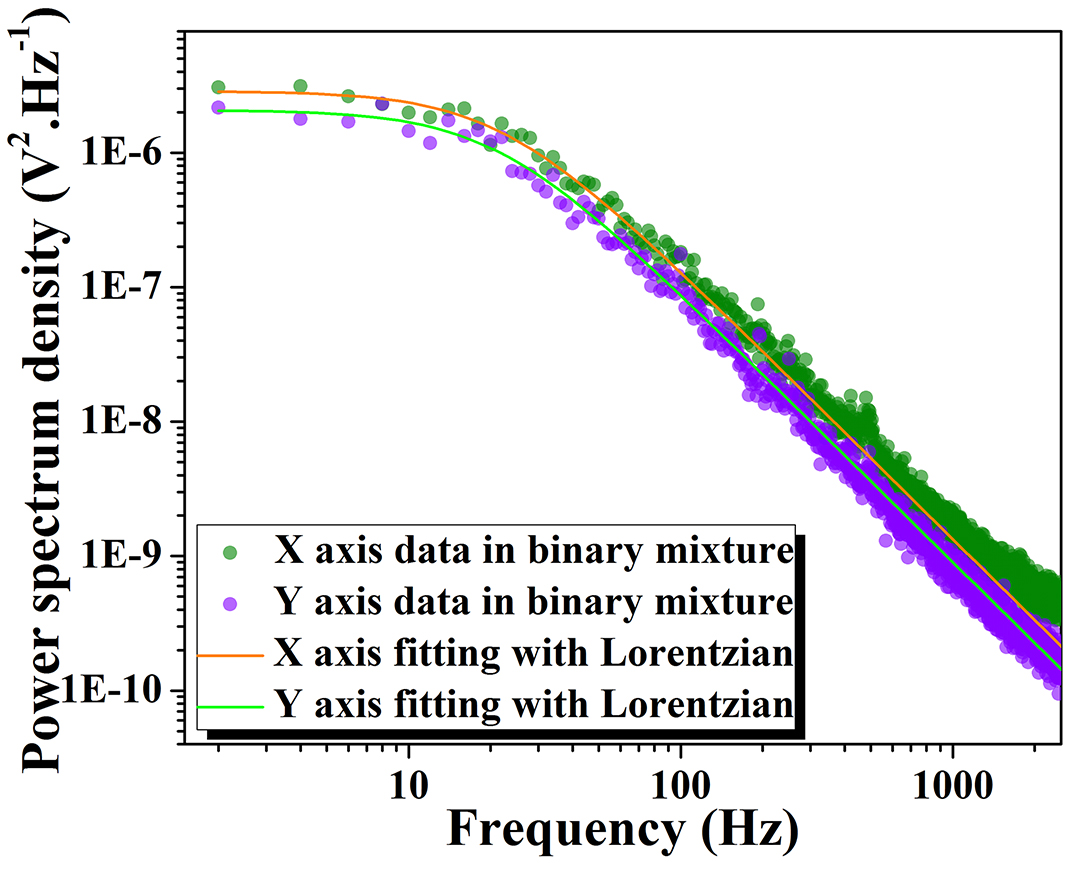

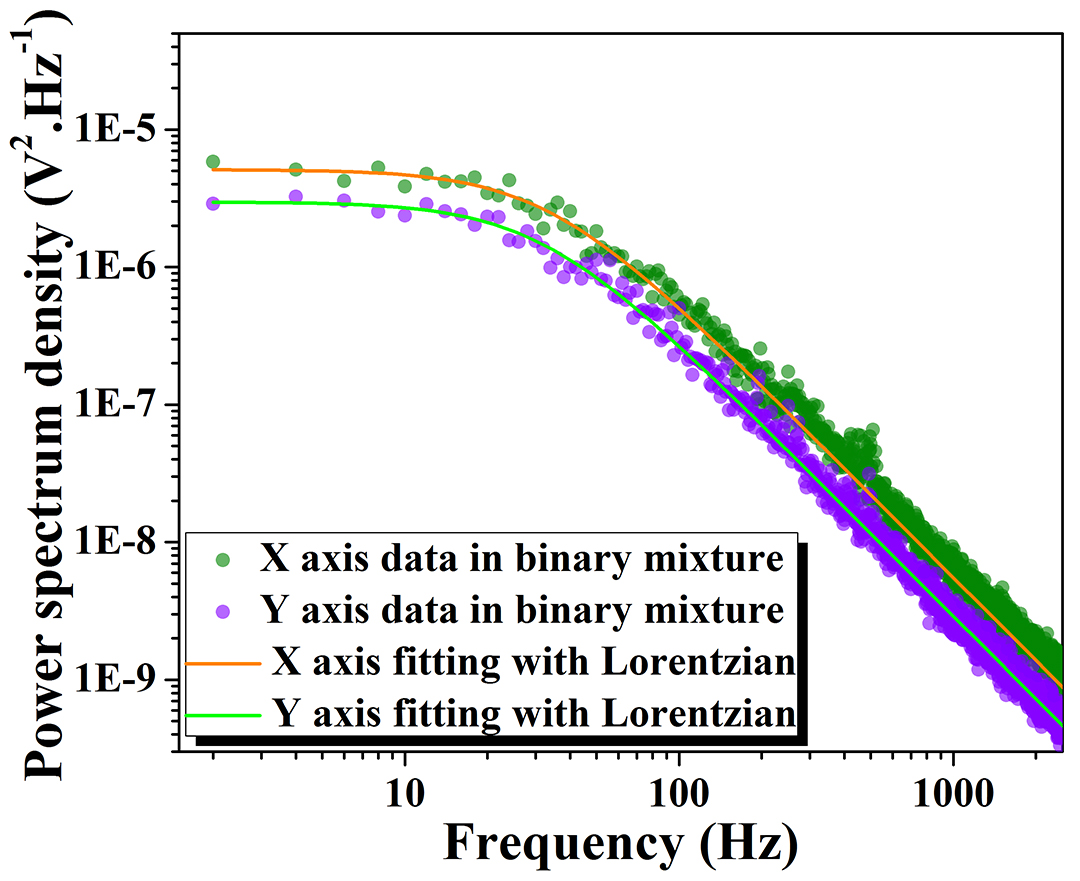

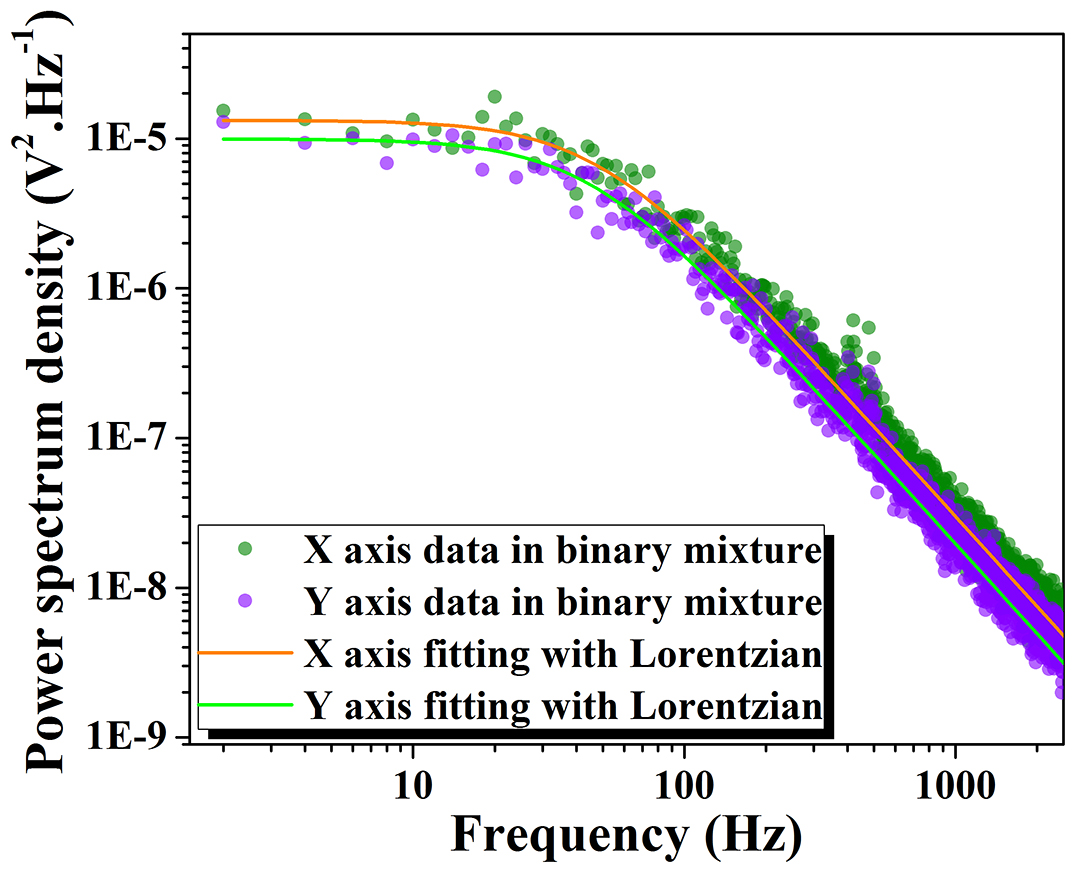

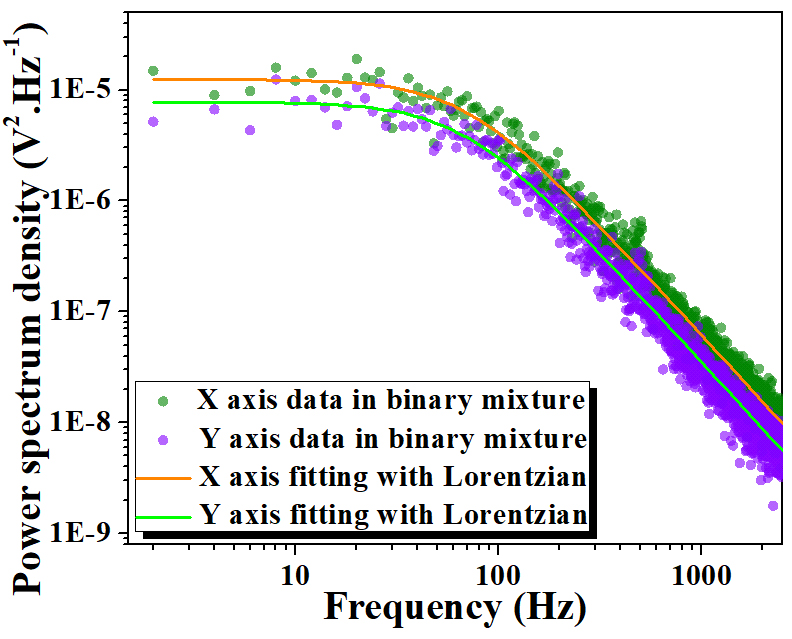

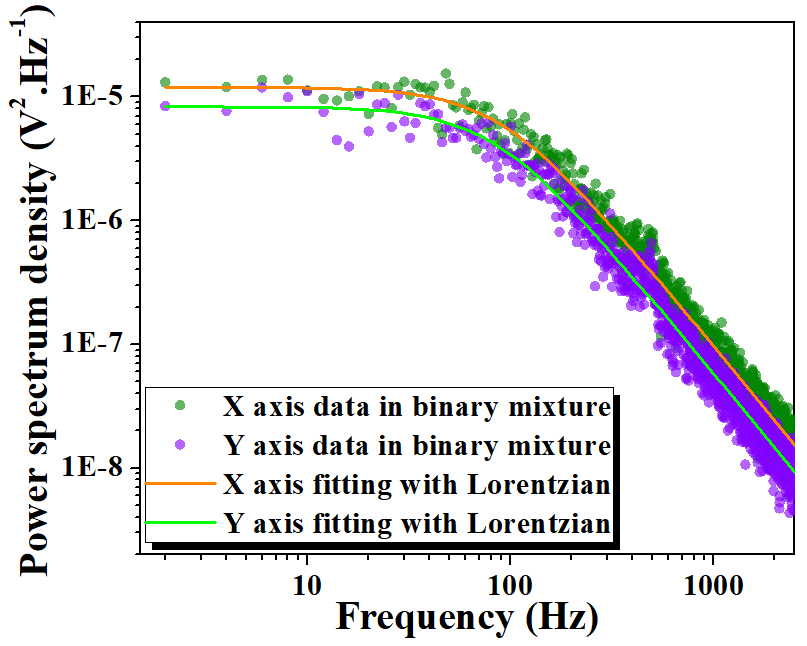

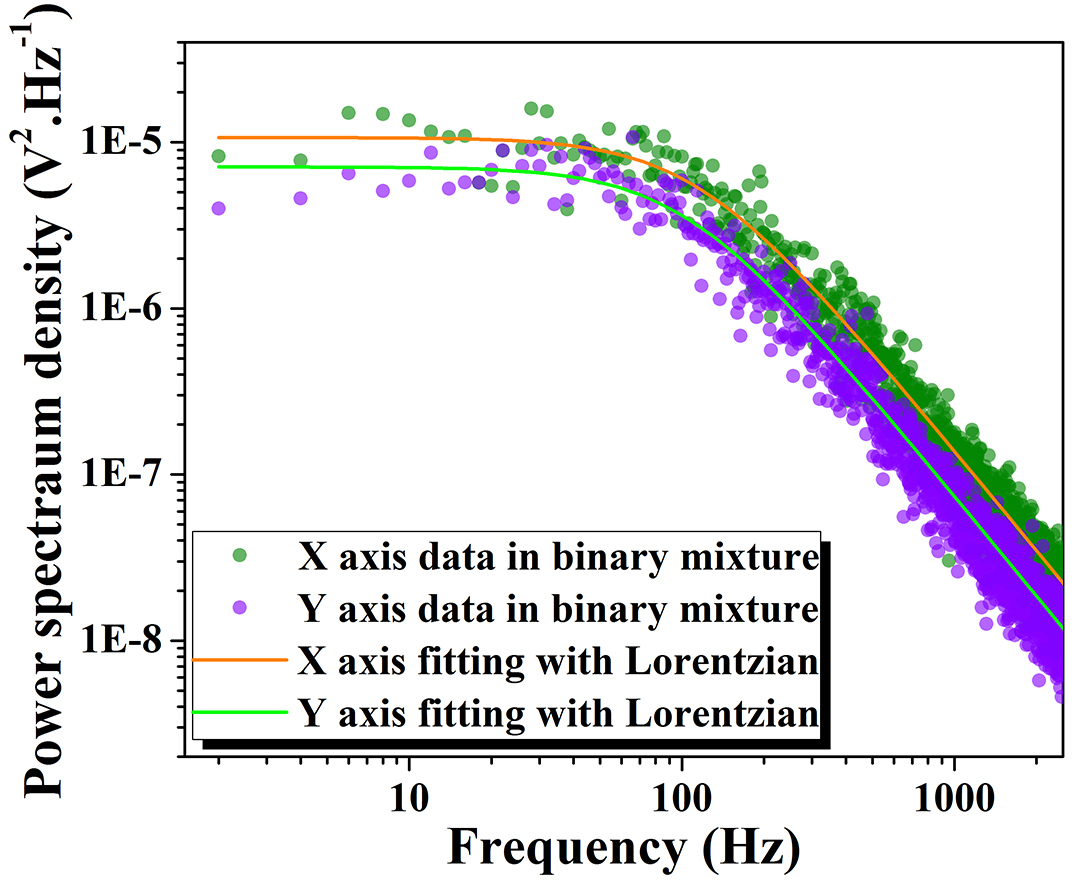


**(f)**

**(a)**

**(e)**

**(d)**

**(c)**

**(b)**

**30 mW**

**25 mW**

**20 mW**

**15 mW**

**10 mW**

**5 mW**


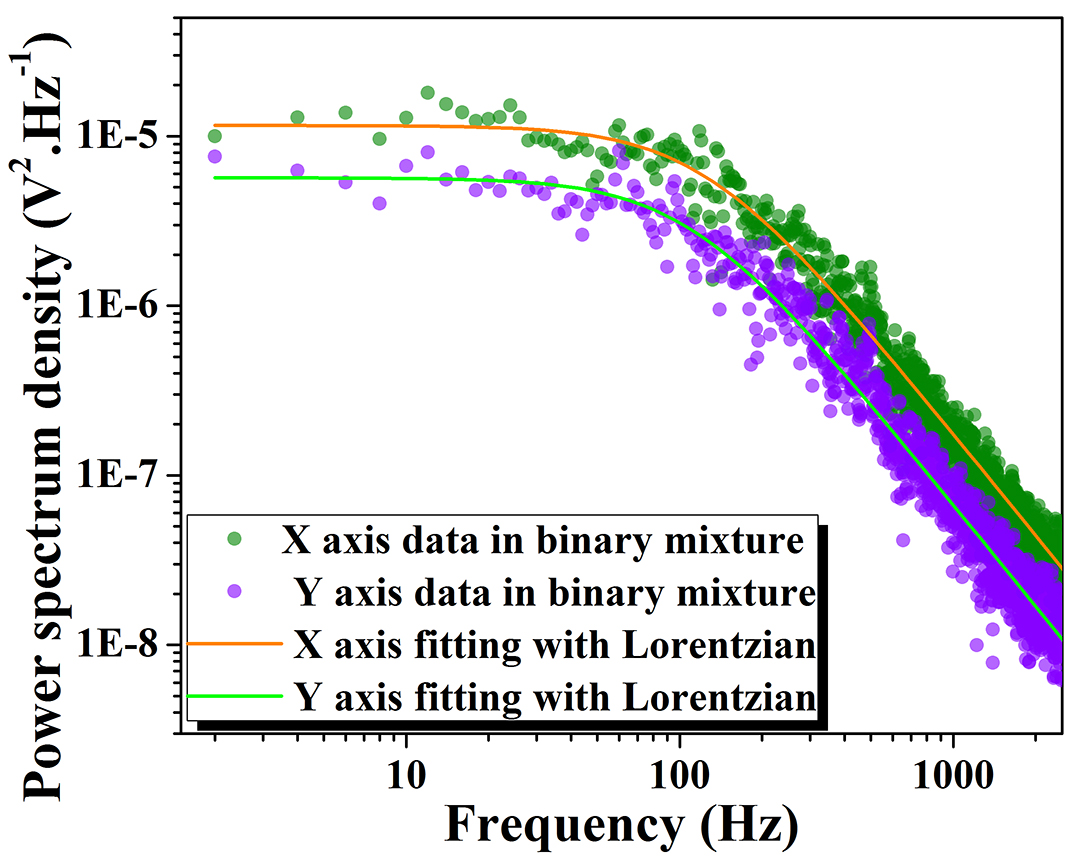




**(h)**

**(g)**

**35 mW**

**Figure 4:** The power spectrum density from trapped particle in 30% Methanol at 5 to 35 mW power (a-g) for both x (olive circle) and y axis (violet circle) and their corresponding Lorentzian fitting (orange line for x axis and green line for y axis). (h) The corner frequency variation for both axis x and y with different average laser power.

**Supporting Information 5**

| **Table 1│** **Comparison of size dependent trap stiffness asymmetry** | | | | | |
| --- | --- | --- | --- | --- | --- |
| **Solvent**  **used** | **Trapping bead radius (nm)** | **f_x_**  **(Hz)** | **f_y_**  **(Hz)** | **η**  **(mPa.s)** | **Experimental**  **κ_asym_= (1-κ_x_/κ_y_)** |
|  | 250 | 227±4 | 168±3 |  | -0.35 |
| Water | 550 | 163±10 | 123±3 | 0.894 | -0.33 |
|  | 1000 | 40±0.5 | 36±0.5 |  | -0.11 |
|  | 250 | 233±8 | 188±6 |  | -0.24 |
| 80% | 550 | 159±3 | 129±2 | 0.872 | -0.23 |
| MeOH | 1000 | 48±1 | 42±1 |  | -0.14 |
|  | 250 | 354±8 | 235±5 |  | -0.51 |
| MeOH | 550 | 239±14 | 166±2 | 0.600 | -0.44 |
|  | 1000 | 58±2 | 48±1 |  | -0.21 |

**Supporting Information: 6**

**
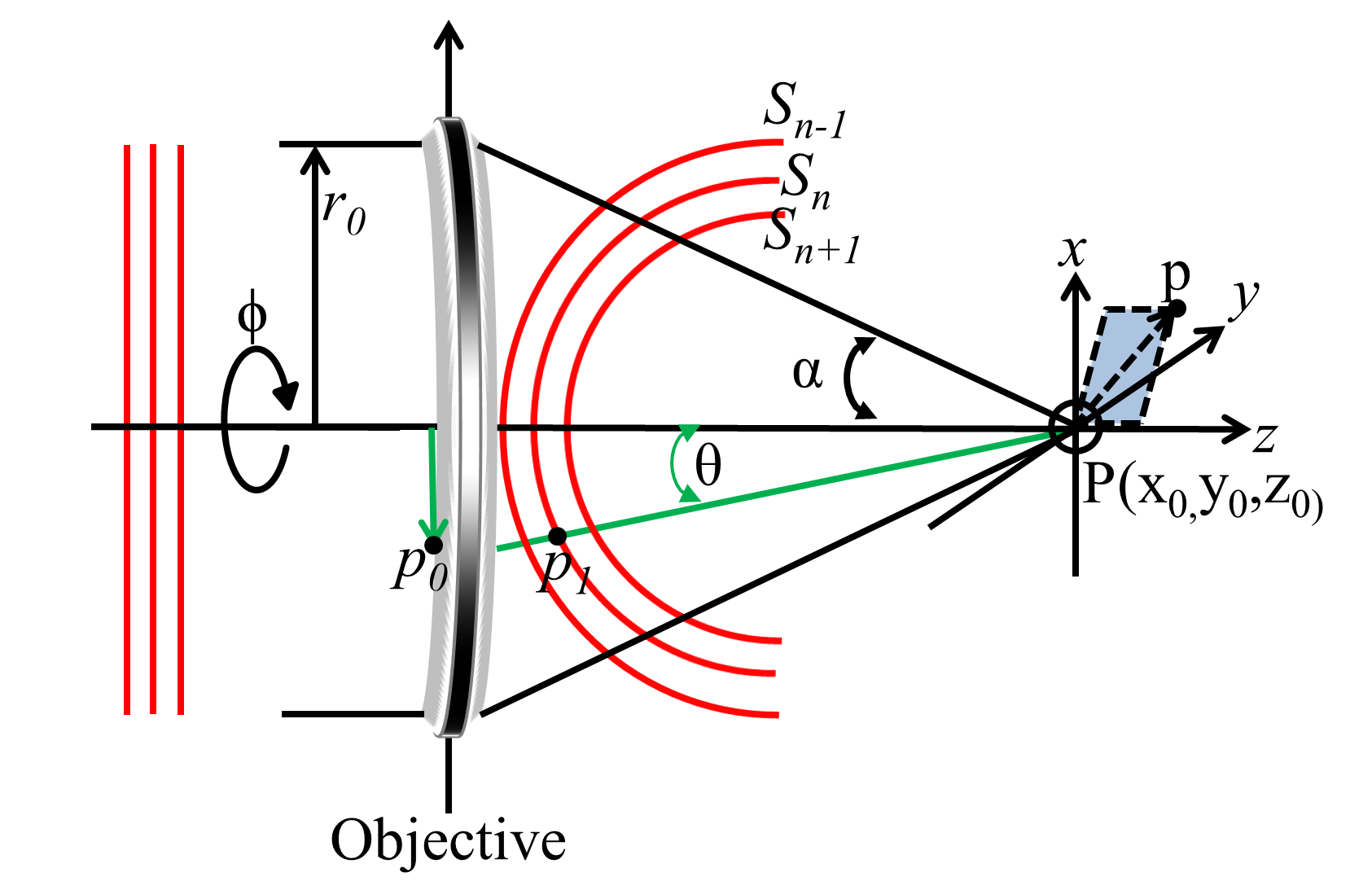
**

**Figure 5A:** Geometrical representation of the propagation of light through a high NA OL. Linear wavefronts transform to spherical wavefronts (S_n­_) after it passes through objective and focus to the point P. A diffraction picture at any point ‘p’, in close vicinity of the focal spot, is given by the Debye-Wolf integral. Maximum focusing angle is α of the NA OL, with the relationship $0<\theta\leq\alpha$ and ϕ is the azimuthal angle of the object plane.


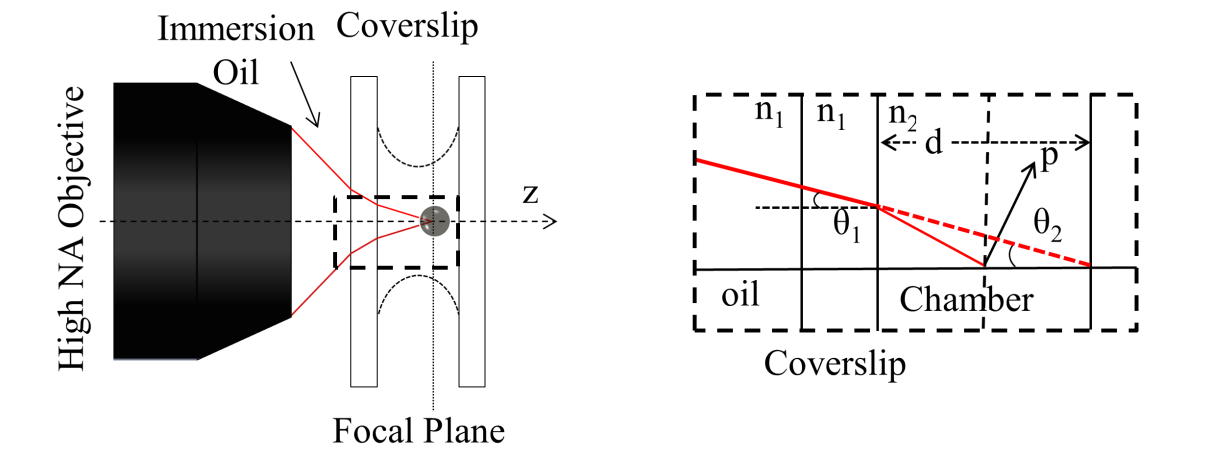


**Figure 5B:** Schematic illustration of the propagation of a tightly focused beam in the presence of refractive index mismatch in the propagation path. In our system the immersion medium and coverslip has same refractive index. They are considered as n_1_ refractive index medium. Water medium is considered as second refractive index medium, n_2_. If the medium was homogeneous (n_1_=n_2_ case), the geometrical focal point would have been ‘d’ distant apart from the starting point of the second medium.

**Supporting Information: 7**

φ

E_r_

dΩ=2πsinθdθ

**Figure 6:** Diagram of the movement of a dipole under polarized light.

**Supporting Information: 8**


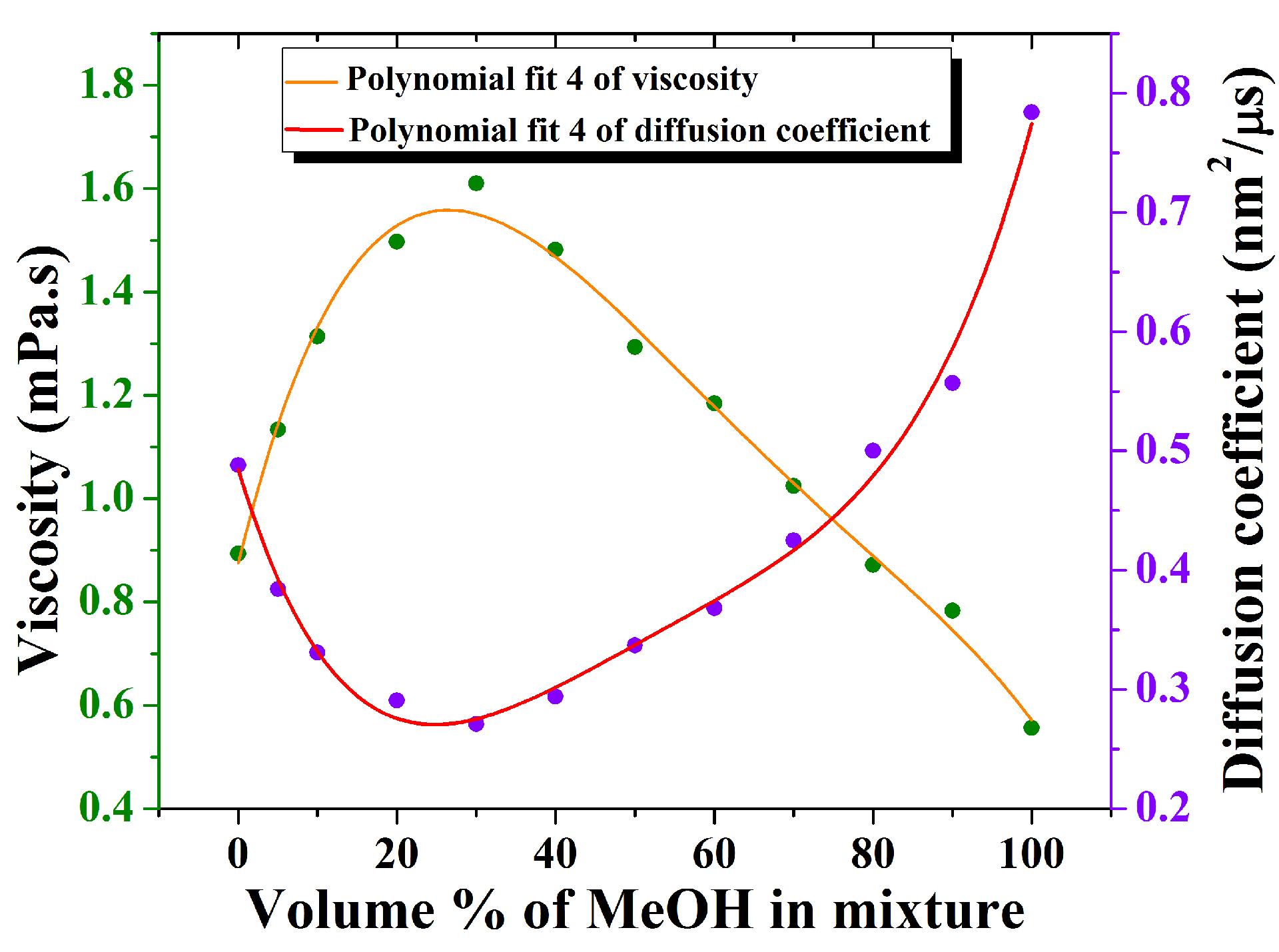


**Figure 7:** Experimentally measured Viscosity (green circle) and diffusion coefficient (purple circle)

**Supporting Information: 9**


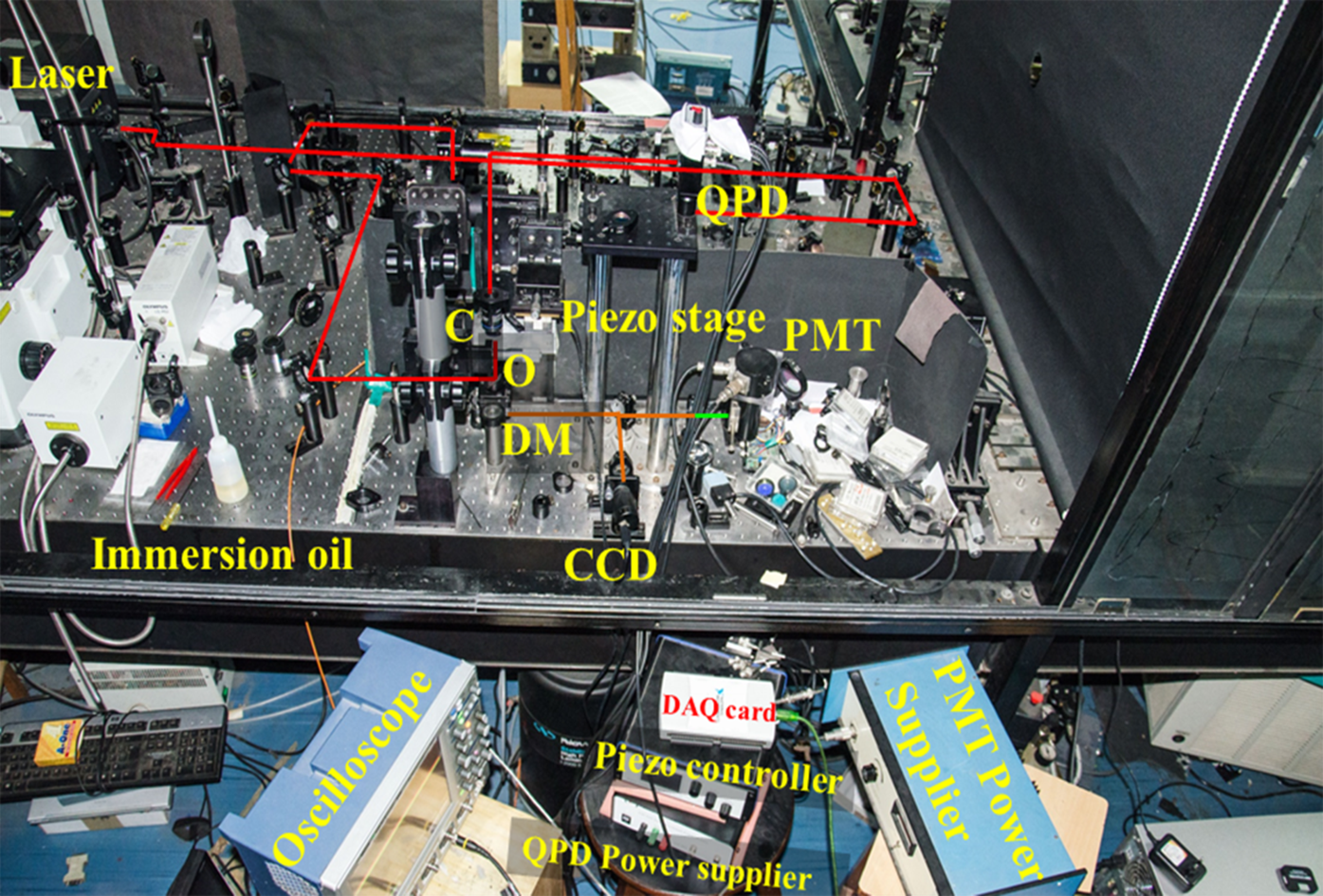


**Waveplate**

**Figure 8:** Images of our experimental setup

**Supporting Information: 10**

**Data analysis procedure:**

Our data analysis procedure is based on the references 29 and 34 in the main manuscript. We have analyzed our data by using power spectrum method, developed by Tolić-Nørrelykke et al. in Rev. Sci. Instrum. 77, 103101 (2006), which utilizes the calibration at back focal plane. In power spectrum, the power is measured in the interval f and f+df, which does not distinguish between +f and –f. In such cases, it is possible to define one sided power spectral density (PSD) by following equation

Before analyzing the experimental data, we have decorrelated our data along x and y axis. We have applied the method which has been introduce and explained by Berg-Sørensen et al.in Rev. Sci. Instrum. 75, 594 (2004) by using our MATLAB programme for this decorrelation method. The MATLAB code is written by following the references 29, 33 and 34 in the main manuscript. For the decorrelation, we have transformed our experimental data to a new linear coordinate system, where in this transformed frame of reference P_xy_ vanishes (basically x and y axis data decouples in this new reference frame), where

For linear transformation, we have chosen two constants, b and c, as described by Kirstine Berg-Sørensen et al. to show that the following criteria is fulfilled

The calculation of b and c in this method will result in the uncouple-ordinate (x', y').

For absolute calibration purposes, a *sinusoidal response function* with frequency f_piezo_ =50 Hz and amplitude A =178 nm is applied throughout our experimental measurement to the piezoelectric stage in X direction. Finite time measurement with discrete Fourier transform function has the expectation value for the one-sided PSD in the following form

Here D is diffusion coefficient. The measured power spectrum in volts is converted to position in the X direction using following equation

Here β is estimated in nm/mV, P is actual height of spike after eliminating thermal background due to Dirac delta function: δ(f-f_piezo_) experimentally created and t_msr_ is 0.5 second in our experiment. Our experimental data is fitted with the first term of the right hand side of the eqn. 5.


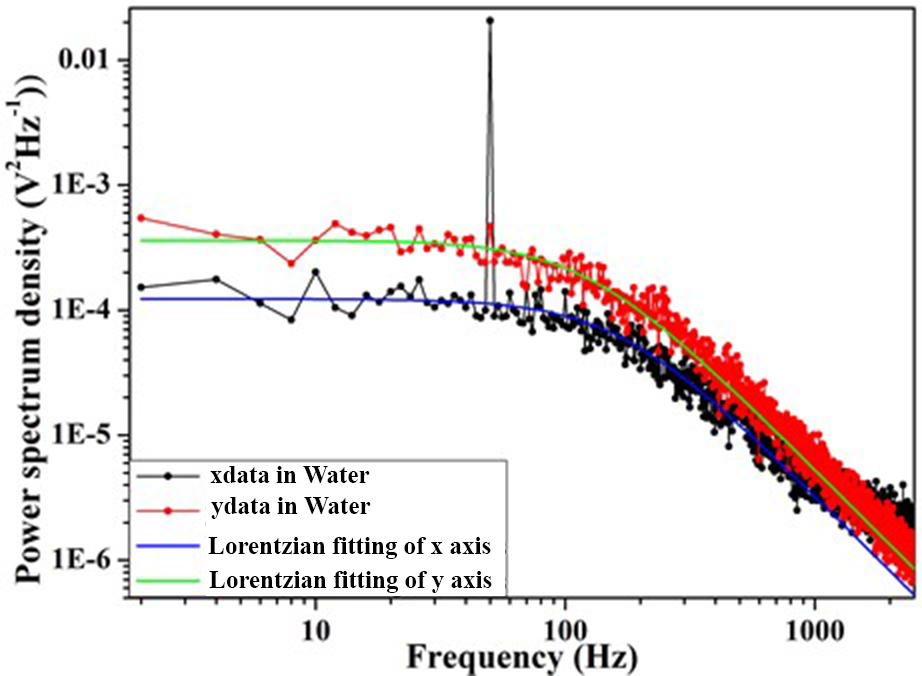

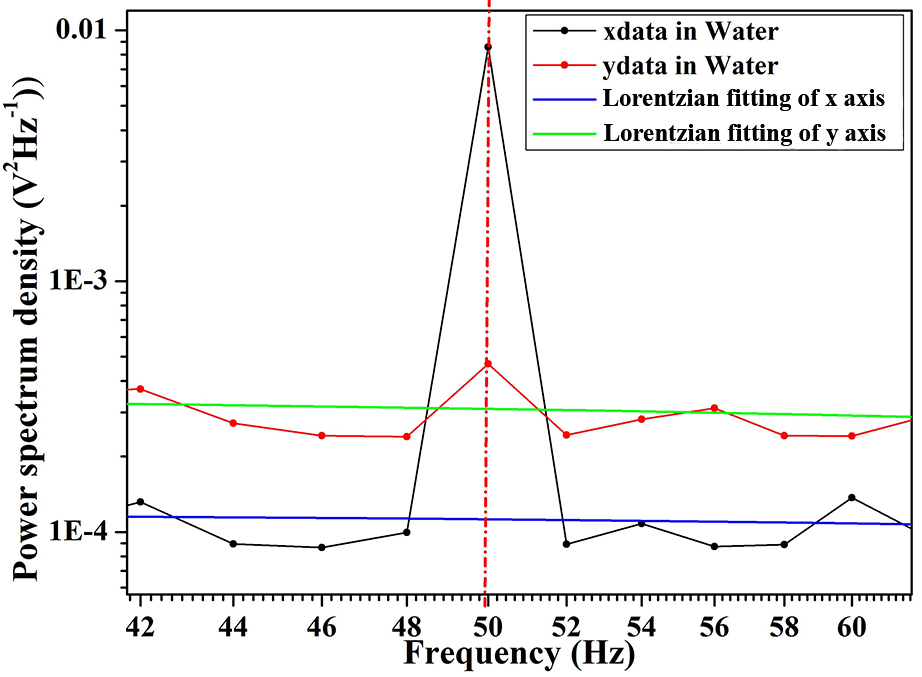

**(b)**

**(a)**

**Figure 9:** power spectrum density (a) Scatter points of xdata (black) and ydata (red) and respective Lorentzian fitted data (Solid line) for 550 nm radius fluorophores coated polystyrene bead in Water. (b) Zoomed part around 50 Hz regions to indicate delta function FWHM.
